# Specificity and diversity of the mouse Peyer's patch mononuclear phagocyte system

**DOI:** 10.18632/oncotarget.4481

**Published:** 2015-06-15

**Authors:** Johnny Bonnardel, Clément Da Silva, Hugues Lelouard

**Affiliations:** Centre d'Immunologie de Marseille-Luminy, Aix Marseille Université UM2, Inserm, U1104, CNRS UMR7280, 13288 Marseille, France

In mammals, gastrointestinal mucosa represents the largest surface of interaction with the external environment. This allows an appropriate absorption of nutrients, electrolytes and water but, as a consequence, the gut is regularly exposed to environmental threats and injuries through ingestion of contaminated food or drink. Thus, it has to deal with a great variety of pathogens. Penetration of these pathogens is normally prevented by different mechanisms including physical barriers (mucus, glycocalyx and tight junctions of the epithelium) and antimicrobial agent secretion. However, some pathogens, such as *Shigella*, *Listeria* or *Salmonella* can survive harsh conditions, disrupt the continuity of the epithelial barrier and transit through the epithelium to reach interstitial tissues. The gut is also densely populated by an innocuous commensal flora which, in addition to participating in the absorption of essential nutrients, plays a crucial role in the detoxification of noxious compounds, protection of the epithelium against pathogens and inflammation dampening. Thus, this microbiota has to be preserved but also tightly regulated to avoid uncontrolled growth of pathobionts. Therefore, the protection ensured by the mucosal immune system is crucial for the organism integrity and a fine-tuned balance between the induction of immune responses to harmful pathogens and the maintenance of tolerance to food antigens and regulation of commensal flora has to be found. The interplay between the mucosal immune system and the microbiota is essential for the preservation or recovery of gut homeostasis. Disruption of this mucosal immune and microbial equilibrium can lead to the development of diseases including severe infection, inflammatory bowel disease, food allergy and cancer. Thus, there are accumulating evidence linking dysbiosis to carcinogenesis. Microbiota disturbance can notably influence cancer development by perturbing the mucosal immune system [1].

Ideally the mucosal immune system should be warned of the presence of pathogens before they manage to penetrate the epithelial barrier. One way to achieve this is to provide an open access of the gut luminal content towards the mucosal surface at very restricted sentinel sites scattered along the gastrointestinal tract. Peyer's patches (PPs) are indeed primary antigen sampling and inductive sites for the establishment of mucosal immunity distributed at key locations of the small intestine. They comprise clustered domes formed by B-cell follicles separated from each other by interfollicular regions enriched in T cells. The follicle-associated epithelium contains specialized epithelial cells, called M cells that bind and rapidly transport antigens from the lumen to the subepithelial dome, where their uptake, degradation and presentation by mononuclear phagocytes, i.e. macrophages and dendritic cells (DC), are key steps to induce the mucosal immune response [2]. It is thus important to clearly define PP mononuclear phagocytes, in order to understand their interplay with the microbiota and the initiation of mucosal immune response against pathogens. Although five mouse DC subsets have been described in PP, little is known about macrophage diversity [3-5]. Moreover, recent studies (reviewed in [6]) have pointed out the substantial overlap in several key surface markers between macrophages and DCs (e.g. CD11c, CD11b, SIRPα, CD68 and MHC-II) leading to identity confusion. Finally, each dome of one PP is surrounded by villi, thus preventing to easily discriminate phagocytes extracted from the domes and those obtained from dome-associated villi (DAV). In our study, we succeeded in distinguishing mouse conventional DC, monocyte-derived DC and macrophage subsets of the dome from those of the DAV [7]. Unlike villus and DAV ones, dome monocyte-derived cells express high amount of lysozyme and lack most of the classic intestinal macrophage markers (i.e. F4/80, CD64, CD169 and CD206). However, monocytes differentiate locally into CD4+ cells that display the characteristics of macrophages, i.e. long-lived cells with strong phagocytic activity but poor naïve T cell priming ability. Interestingly, monocytes also give rise to CD4- lysozyme-expressing DC (LysoDC) which, unlike dome macrophages, display a rapid renewal rate, strongly express genes of the MHCII presentation pathway and efficiently prime naïve helper T cells for IFNγ production. LysoDC and macrophages share however many common features such as particulate antigen uptake, strong innate antiviral and antibacterial gene signatures and, upon TLR7 stimulation, IL-6 and TNF secretion.

In summary, our results show that in PP monocytes develop into two closely-related cell types with different lifespan and functional properties. These monocyte-derived cells differ greatly from their villous counterparts pointing out the important variation between the microenvironment of gut immune inductive (i.e. PP) and effector sites (i.e. villus *lamina propria*). Thus, the dome microenvironment exerts a strong influence on the differentiation program of phagocytes. This specialization likely contributes to the crucial role of PP in the mucosal immune response induction and future studies of specifically expressed genes will probably help to understand the mechanistic and pathways involved in this process.
